# Planning for successful participant recruitment and retention in trials of behavioural interventions: Feasibility randomised controlled trial of the Wrapped intervention

**DOI:** 10.1371/journal.pdig.0000875

**Published:** 2025-05-29

**Authors:** Lauren Schumacher, Rik Crutzen, Kayleigh Kwah, Katherine Brown, Julia V. Bailey, Stephen Bremner, Louise J. Jackson, Katie Newby

**Affiliations:** 1 Public Health and Applied Behaviour Change (PHAB) Lab, University of Hertfordshire, Hatfield, United Kingdom; 2 Department of Health Promotion, Care and Public Health Research Institute (CAPHRI), Maastricht University, Maastricht, The Netherlands; 3 eHealth Unit, Department of Primary Care and Population Health, University College London, London, United Kingdom; 4 Department of Primary Care and Public Health, Brighton and Sussex Medical School, Brighton, United Kingdom; 5 Institute of Applied Health Research, College of Medical and Dental Sciences, University of Birmingham, Birmingham, United Kingdom; Iran University of Medical Sciences, IRAN, ISLAMIC REPUBLIC OF

## Abstract

Randomised controlled trials (RCTs) must have sufficient power if planned analyses are to be performed and strong conclusions drawn. A prerequisite of this is successful participant recruitment and retention. Designing a comprehensive plan for participant recruitment and retention prior to trial commencement is recommended, but evidence concerning successful strategies, and how to go about developing a comprehensive plan, is lacking. This paper reports on the application of a six-stage process to develop a recruitment and retention strategy for a future RCT. Stage 1) Rapid evidence review: strategies used in previous trials were identified through database searching. This informed Stage 2) PPI workshop: workshops with public and patient involvement (PPI) group were used to select a sub-set of these strategies based on their potential to be successful and acceptable with the target audience. Stage 3) Focus groups with the target audience: the sub-set was refined through feedback from 15 young people (data subjected to content analysis). Strategies the PPI and focus groups mutually agreed upon proceeded directly to Stage 5; those without consensus proceeded to Stage 4. Stage 4) PPI workshop: PPI members voted on the remaining strategies; those without consensus were discarded. Stage 5) Observation of strategies during feasibility RCT (fRCT): the retained set of strategies were observed in practice in a fRCT in which recruitment and retention data and qualitative feedback from participants was collected. Stage 6) PPI workshop: the fRCT findings were reviewed and strategies for use in the future RCT were finalised. The finalised strategy included set of adverts; schedule of financial incentives; instructions to send survey invite by email, one prompt by SMS prior to data collection, and up to three SMS reminders; procedure to keep participants engaged (e.g., newsletters, personalisation of communications); and procedure if participants fail to complete a research activity (follow-up email/phone call).

## Author summary

To draw firm conclusions, all randomised controlled trials (RCTs) must successfully recruit and retain participants. We followed a six-stage process to develop a recruitment and retention strategy for a future RCT. This involved a rapid review of evidence to identify strategies used by other randomised controlled trials (stage 1), four workshops with a public and patient involvement (PPI) group who discussed and refined the strategies (with the target sample in mind) and then cast votes to determine which should be taken forward (stages 2, 4 and 6), focus groups with the target sample (15 young people aged 16–24 years) to get direct feedback on evolving strategies (stage 3), and observation of the strategies during a feasibility RCT (stage 5). Our finalised strategy set out the recruitment adverts to be used (highlighting the value of participation, e.g., financial reward), methods for keeping participants engaged (e.g., personalisation, newsletters), the schedule of voucher payments, the frequency and mode of data collection invites and reminders (one invite, up to three reminders, email and SMS), and actions to minimise non-response (email/telephone follow-up). The process we took, and the strategies produced, may be of interest to others preparing to run a RCT.

## Introduction

The success of randomised controlled trials (RCTs) rests on recruiting sufficient participants and retaining them for the duration of the study, both of which can be challenging [1,2]. If recruitment is not completed at the expected rate, if it fails to produce the required sample, or if there is loss of participants during the study, it can have knock-on costs to the project in terms of end-date and budget [3], as well as resulting in insufficient power to draw valid conclusions [1]. Furthermore, attrition often causes bias, with loss typically being patterned; for example, attrition is more likely from the intervention arm than the control arm in health behaviour change trials [4], and among groups with specific demographic characteristics, such as from younger age groups [5], male participants [6], and those with lower levels of education [7]. The importance of these challenges is reflected in the results of a Delphi study with Clinical Trials Unit Directors, who identified that the two most important methodological issues for RCTs were increasing recruitment and retention [8].

Recruitment to trials is a major challenge: a review of trials examining healthcare interventions published between 2004 and 2016 found that only 56% of these met planned recruitment targets [9]. This review also reported retention rates. While these were high, with a median of 89%, the authors advised that this figure should be regarded with caution, due to over a third of included trials featuring short term retention periods of less than 6 months [9]. A further meta-analysis of health behaviour changes trials with a mean follow-up period of 8.1 months reported median retention as 64.5% in the intervention group and 57.5% in the control group [4].

Recruitment and retention can be adversely affected if researchers fail to effectively communicate the opportunity, relevance, and expectation (burden) of participation [10]. Where research examines sensitive issues, such as sexual behaviour, recruitment and retention may also be negatively impacted by heightened concerns around confidentiality and data protection [11], though these challenges may be overcome if the research team is perceived to be from a trusted organisation, such as the NHS or a university [12]. Trial setting can also impact the success of recruitment and retention. Although online trials have the potential to reach large audiences, and to facilitate engagement with research, high dropout is often observed [6,13]. Potential causes of this include participants feeling more anonymous and therefore less committed to the research [14] and requests for follow-up data collection being perceived as spam [15]. Overall, the strategies behind successful trial recruitment and retention are not well evidenced [16], with few trials reporting those employed [17]. What works best for online trials is even less well understood.

A review and meta-analysis by Treweek and colleagues [2] identified strategies associated with successful recruitment. Reminders, financial incentives, and specific trial design features such as the unblinding of participants to allocated treatment, were singled out as potentially effective approaches. They noted however that the evidence for these strategies was limited due to included studies often being small and the presence of significant heterogeneity across studies.

Drafting a plan for successful recruitment prior to study commencement, rather than resolving issues as they arise during the active trial period, is recommended in order to maximise recruitment and efficiency of the research team [18]. The Clinical Trials Transformation Initiative created a framework to guide researchers in developing effective recruitment strategies. This framework includes the following steps: 1) Trial Design and Protocol Development, (optimising data collection and study design, with input from key stakeholders); 2) Trial Feasibility and Site Selection, (understanding the context of recruitment and planning for maintaining and evaluating active recruitment); and 3) Recruitment Communication Planning, (developing a strategy for effectively engaging stakeholders). The framework also advises that successful trial recruitment plans should involve various stakeholders as applicable to the trial context, such as public and patient involvement (PPI) groups [18]. While the framework provides useful guidance it does not provide detail on how to perform these steps, nor does it address how to maximise retention; the authors argue that activities to improve recruitment will also improve retention [18]. Though recruitment and retention are related, they are separate issues that at times require different approaches. The present study provides the required detail to perform step 1 ‘Trial Design and Protocol Development’, describing the co-development of a trial recruitment and retention strategy with a PPI group.

The present study was conducted as part of a feasibility randomised controlled trial (fRCT) that aimed to assess whether and how it was possible to carry out a future definitive RCT of a digital sexual health intervention called Wrapped [19]. The online fRCT aimed to recruit 230 UK based 16–24 year old users of a sexually transmitted infection (STI) testing website and retain a minimum of 60% at the 12 month follow-up. Data was collected at baseline and months 3, 6, and 12 via online surveys and postal STI test kits. For more detailed information of the intervention content and fRCT design see Newby et al. 2019 [20] and Newby et al [19] respectively.

Prior to initiating the Wrapped fRCT, a comprehensive strategy was developed to support successful achievement of the fRCT’s primary recruitment and retention objectives. This paper aims to describe the novel and detailed process through which this strategy was developed and offers an example of how this process can be used in other trials as well. This paper, to the best of our knowledge, for the first time describes detailed guidance on how to develop a strategy to maximise trial participation.

## Methods

### Ethical approval

Ethical approval was granted by the Health, Science, Engineering and Technology Ethics Committee with Delegated Authority (ECDA) at the University of Hertfordshire (reference LMS/SF/UH/04061). Formal consent was obtained for all participants via REDCap, a secure data capture and management platform [21,22].

### Design

**Fig 1:**
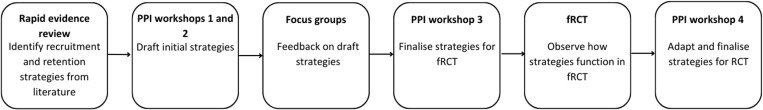
Development of recruitment and retention strategies.

The recruitment and retention strategy was developed iteratively across six stages. These encompassed, 1) Rapid evidence review, 2) PPI workshops 1 and 2, 3) Focus groups with target audience, 4) PPI workshop 3, 5) Observation of strategies during fRCT, and 6) PPI workshop 4; see Fig 1. More detail on each stage is provided below.

### The Patient and public involvement (PPI) group

The PPI group was formed of 6 members (2 female, 4 male) aged 20–24 years old, all of whom were users of freetest.me (an online chlamydia self-sampling service operated by Preventx Ltd. which is commissioned by local authorities in England and free at the point of use). All members identified as White ethnicity; information on sexual orientation was not collected. The group was initially recruited to assist in the co-development of the Wrapped intervention using an advert placed on freetest.me. The group went on to support preparation of the Wrapped fRCT funding application and then to continue their involvement in the research upon study commencement.

The PPI group collaborated with the research team to provide the perspective of the target population, to ensure that the research was acceptable and understandable to participants, and to help create a positive research experience for participants.

### Procedure

#### Stage 1: Rapid evidence review.

The aim of the rapid evidence review was to identify strategies employed by RCTs testing a digital intervention that achieved relatively high recruitment and/or retention rates. PubMed, Scopus, PsycArticles, CINAHL and Cochrane databases were searched in May 2020. Keywords were combined to capture different types of studies that assessed a digital intervention: (“digital intervention” AND (RCT OR “randomised controlled trial” OR “randomized controlled trial” OR fRCT OR review OR “feasibility study”)); searches were not restricted by publication date. Articles were considered eligible if the study was a RCT, fRCT, review, or theory paper reporting recruitment and/or retention rates, as well as any strategies used to obtain adequate recruitment and retention in the assessment of a digital intervention. Articles were considered ineligible if they were protocol papers or conference proceedings.

Duplicates were eliminated using Mendeley [23] and titles and abstracts were screened for eligibility. Full text articles were obtained for articles that appeared to meet inclusion criteria. The following data was extracted for included articles: study design, population, recruitment rates (rate or proportion of sampling pool recruited) and strategies, any recruitment messages used to advertise the study, retention rates (percentage of participants completing the final data collection time point) and strategies, and any qualitative feedback. Strategies were organised thematically into categories as follows: value propositions (statements which communicate what individuals can gain from participation), engaging participants with the research (strategies used to develop and maintain participant engagement), nature of survey invitations, value and implementation of financial incentives, reminders to complete activities, and actions to minimise non-response. All categories were developed inductively over the course of the analysis.

As this was a rapid evidence review, all searches, selection for inclusion, data extraction, and analysis was conducted by a single author (LS). The quality of included studies was not assessed.

#### Stage 2: PPI workshops 1 and 2.

The aim of this stage was to draft initial strategies with the PPI group. The group first met online (via Microsoft Teams) [24] with a researcher for an ice-breaker session at which the aims of the study and the role of the PPI group were discussed. The intention was to follow this up with two face-to-face workshops at which a set of provisional strategies would be developed. In light of the COVID-19 pandemic however, this plan was changed. The group instead decided to work together asynchronously on a shared workspace called Padlet [25], a cloud-based application that allows users to create, share and organise material together. Two Padlets were used, the second of which was informed by the first. Five members contributed to each Padlet, both of which were made available for the group to complete over a one-week period.

In the first Padlet, material was presented in columns, and members were instructed to work across each column in turn. Each column presented a different recruitment and retention category identified from the rapid review (i.e., value propositions, retention, reminders, etc.). Members were first asked to give their own ideas for strategies against each category, before moving to subsequent columns where they learned what specific strategies had been identified from the rapid evidence review and were asked to provide feedback on each one. For example, for retention strategies participants were first asked to provide their own ideas on how the research team could best encourage participants to complete all data collection points over the course of the study; members used free text boxes to post their suggestions. Then the strategies identified from the rapid review were presented; members were asked to provide feedback in free text boxes on each strategy and to identify how each strategy might be perceived by them personally or someone in their age range, and if/how that strategy might have negative consequences for participants or the research team. PPI members also used free text boxes to develop potential adverts; similar to the example above, members were first asked to develop their own adverts based on what they thought would be effective; then the strategies identified from the rapid review were presented for their feedback. In addition to the free text boxes, members were asked to indicate their like or dislike for the strategies identified from the rapid review by clicking either “thumbs up” or “thumbs down” on them.

On completion of the Padlet, the research team collated the ideas and feedback received and used this to create a second Padlet. At this point the research team also drafted some further provisional adverts and added these to the Padlet. This second Padlet was presented back to PPI members who were asked to vote on the refined set of strategies and the set of adverts. Strategies receiving the lowest votes or with multiple negative comments did not advance to Stage 3. Completed Padlets from workshops 1 and 2 are available on an open-access basis from the University of Hertfordshire Research Archive [26]. A draft version of the recruitment and retention strategy was created upon completion of stage 2.

#### Stage 3: Focus groups with the target audience.

The aim of this stage was to obtain feedback on the draft version of the strategies, utilising empirical data collection from three focus groups. Participants were 15 young people aged 16–24 years recruited from one of three sources: undergraduate psychology department (n = 8), an organisation working with young people in or leaving the care system (n = 2), and a youth theatre organisation (n = 5). Participants were aged 18–22 and predominately female (n = 13); participants described their ethnicity as White, Black, Asian, and Multiple ethnic background. For more details, see demographics table in S1 Table. Participants were required to give informed consent before taking part (with parental consent additionally required for any individuals under the age of 18 years).

All focus groups were held virtually through Microsoft Teams and were audio and video recorded. Focus groups lasted between 44 minutes and 103 minutes (mean 69 minutes). See S2 Table for the focus group schedules. All group discussions were facilitated by the first author (LS). Two of the three groups were also co-facilitated by a PPI member who had received training from the research team on facilitating focus groups. All recordings were transcribed verbatim, after which the original recordings were destroyed. Focus groups began with an introduction to the fRCT and an explanation of the aims of the focus group session.

Focus group discussions were guided by a Padlet (shared with attendees during the session), which displayed the draft recruitment and retention strategies, along with additional ideas and prompts added by the research team where further input was required. Discussions moved through each category, with participants encouraged to verbally share their views on each one, reflecting on how successful or unsuccessful each strategy or potential advert might be, as well as whether there might be any unintentional negative consequences. Once discussion of each category was completed, participants were asked to vote (using either “thumbs up” or “thumbs down”) on the Padlet to indicate which ideas they thought would be most successful. Participants also left comments in free text boxes on the Padlet with their opinions and suggestions. An example of a completed Padlet from Stage 3 is available on an open-access basis from the University of Hertfordshire Research Archive [26].

The focus group transcripts and comments left on the Padlets were analysed by a single author (LS) using content analysis, with a directed, deductive approach [27]. Analysis was guided by the aim of discovering which recruitment and retention strategies would be the most and least successful, with the analysis focusing on each category discussed in the focus group: value propositions (adverts), engaging participants with the research, nature of survey invitations, value and implementation of financial incentives, reminders to complete activities, and actions to minimise non-response. A predefined Excel spreadsheet was used for each category, with tabs for each potential strategy or advert. Columns were used on each tab to classify each participant’s opinions on the positive, negative, or neutral impact that each strategy or advert might have on recruitment and retention within the trial; quotes were added in rows corresponding to each column. The number of “thumbs up” and “thumbs down” votes was also recorded alongside the data.

#### Stage 4: PPI workshop 3.

The aim of this stage was to finalise the recruitment and retention strategies for the fRCT. The research team examined the analysis and votes from Stage 3 to identify all strategies with mutual agreement between the PPI group at Stage 2 and focus group participants at Stage 3; those with agreement were considered final and automatically added to the final strategy document. The remaining strategies were presented in a Padlet to the PPI group who were asked to provide their feedback within a week. As some parts of the adverts were preferred over others, the research team presented both the complete adverts from Stage 3 as well as their component parts back to the PPI group; these component parts included a call to action (e.g., “we need your help!”), a description of the study, and mention of the incentive to join (such as financial incentives). Three PPI members completed this Padlet. The completed Padlet from Stage 4 is available on an open-access basis from the University of Hertfordshire Research Archive [26].

In creating the final recruitment and retention strategy document, the research team tallied all votes and considered all comments and feedback from the Padlet. Where the PPI group was divided on a strategy, the research team (LS, KK, KB, and KN) made the final decision; these decisions were based on previous experience of conducting fRCTs. Adverts with high levels of similarity were combined. The remaining adverts were checked and revised (where required) to ensure that they met HRA Ethics Guidance, specifically that financial incentives should not be described in the first line of an advert [28]. Additionally, two new adverts were drafted by the research team based on the highest voted component parts. Any potential strategies that were not technically achievable (e.g., if it was not possible to implement a given strategy within REDCap) were also removed. At this point, the recruitment and retention strategy for use in the fRCT was finalised.

#### Stage 5: Observation of strategies during fRCT.

The aim of this stage was to observe how the recruitment and retention strategy functioned with a fRCT. A fRCT was undertaken from March 2021 to October 2022. Recruitment and retention were monitored throughout the trial. During delivery, minor adaptations were made to some of the strategies due to unexpected problems or to test out potential new strategies. All changes were logged for future discussion with the PPI group.

#### Stage 6: PPI workshop 4.

The aim of this stage was to adapt and finalise the strategies for a RCT. Upon conclusion of the fRCT, the PPI group met online via Microsoft Teams to discuss the minor amendments that were made to the strategies. Prior to meeting, a final Padlet was designed to update the PPI group on the specific changes that were made to the strategy during the trial; members were asked to read through the Padlet beforehand so that the meeting could focus solely on their feedback. During the meeting members provided verbal feedback on these changes and reflected on any further changes that could be made to refine the trial process for a full RCT; one author (LS) made brief notes during the meeting. Three members completed the Padlet and attended the meeting. Discussions focused on why changes were made and if they were acceptable, how to make further improvements for the full RCT, and what improvements could be made for the management of the PPI group.

The meeting was recorded and transcribed via Microsoft Teams. The transcript was examined to fill in the details from the notes taken from the meeting and participants’ feedback on the strategy amendments was noted down.

## Results

A total of 226 records were identified from the database search. Duplicates (n = 15) were removed, leaving 211 records to be screened by title and abstract; 134 were excluded. This left 77 full text articles to be screened for eligibility. A total of 36 articles, describing a total of 26 studies, met the inclusion criteria and were included for analysis. For a description of articles included in the rapid evidence review, see S3 Table. The following subsections summarise the findings by strategy category. Although the strategies were considered within the context of each study’s recorded recruitment and retention rates, no uniform pattern emerged. As such, all strategies were considered to carry equal weight.

### Value propositions/adverts

#### Stage 1: Rapid evidence review.

Seven studies [29–43] referred to the value propositions that were used to advertise the study to the target population. Value propositions were framed around the themes of altruism (either as the opportunity to help with research or to help others), mentioning financial incentives, improving health and/or wellbeing, scarcity of participation spaces, and emphasis on how taking part was easy. Additionally, it was noted that the included papers advised that value propositions should be created in collaboration with experts and/or the target population and should use professional design and/or logos to indicate that the message is coming from an expert and/or trusted source. These themes were carried through to Stage 2. See S4 Table for a summary of the different value propositions identified with examples, where available.

#### Stage 2: PPI workshops 1 and 2.

In workshop 1, PPI members drafted adverts which they thought would attract people to join the fRCT and to encourage them to continue participating for the entire 12-month trial period. Eight distinct adverts were drafted that appealed to the values of altruism and prioritising sexual health, and which highlighted the financial incentive (members felt that financial incentives would be the most effective way of attracting participants).

In workshop 2, 12 potential adverts were shown to the PPI group: the eight created during the first workshop and an additional four created by the research team based on the findings from Stage 1. Members were asked to select the six adverts that they thought would be the most successful at attracting participants into the study. The six adverts with the highest votes proceeded to Stage 3 for further discussion by the focus group participants; see S5 Table for details of voting and which adverts proceeded to Stage 3.

#### Stage 3: Focus groups with the target audience.

Participants were presented with the six highest voted potential adverts from Stage 2 via Padlet and asked to provide their thoughts and reactions to them. Participants coalesced around several key elements that would make a successful advert:

- Keeping it brief and to the point.
- Mentioning the financial incentive, which was considered the most important motivating factor.
- Appealing to altruism.
- Conveying to participants that they will be valued.
- Indicating that commitment was required, but that taking part was easy.

Two focus group participants drafted their own potential adverts highlighting altruistic and financial rewards (see S6 Table). These, along with two further adverts created by the research team, progressed to Stage 4 for further discussion. For more details on feedback from focus group participants on value propositions, including participant quotes, see S7 Table.

#### Stage 4: PPI workshop 3.

PPI members voted on potential adverts created by the focus group participants and by the research team based on analysis from Stage 3; the four highest voted adverts were selected for inclusion in the final recruitment and retention strategy for the fRCT. PPI members also voted on the component parts of the adverts; the highest voted components were then used by the research team to draft two new adverts. For more details, including the final votes and PPI quotes from the Stage 4 Padlet, adverts and PPI voting outcomes, and PPI voting on advert component parts, see S9, S10, and S11 Tables respectively. Below is the list of final adverts included in the fRCT:

1. Help us make a positive change to young people’s sexual health: make a difference by joining our study and get paid for your time.
2. Take part in a study to improve sexual health and earn up to £65 in vouchers, find out how you can make a difference today.
3. Your views are important: join our study to help improve sexual health for young people while being paid!
4. Make a difference to improve sexual health for young people - join our study and earn up to £65 in vouchers for your time.
5. Want to make a difference to young people’s sexual health? Take part in our study and get paid for your time.*
6. We need your help! Take part in a study to improve young people’s sexual health and earn up to £65 in vouchers for your time.*

*Created from value proposition components

#### Stage 5: Observation of strategies during fRCT.

In order to address lower rates of recruitment, especially among 16–19 year olds, a further advert was created, with a specific variant for participants aged 16–19:

- Interested in sexual health research? Take part and get up to £100 in vouchers.
- Interested in Sexual Health Research and aged 16–19 years? Want to earn up to £100 in Amazon Vouchers?

No discernible differences in recruitment rates in terms of the advert displayed to potential participants was observed in the fRCT.

#### Stage 6: PPI workshop 4.

PPI members supported this additional advert and acknowledged the barriers young people aged 16–19 might have experienced.

### Engaging participants with the research

#### Stage 1: Rapid evidence review.

Seven studies referred to strategies used to maintain engagement in the study [30–33,36–38,44–50]. Strategies included:

- Maintaining continuity throughout all participant materials (continuous branding in the form of logos, colours, fonts, templates, and formatting).
- Personalising all communications (i.e., addressing participants by name).
- Communications kept brief, clear, and simple.
- Social desirability statements used in communications.
- Communications with intervention group to remind them to access the intervention.
- Thanking participants for either joining the research or continuing to participate.
- Updating participants on the progress of the research via newsletters.

These themes were carried through to Stage 2

#### Stage 2: PPI workshops 1 and 2.

The PPI group felt it was important for retention that participants knew that their input was valued by the research team. In this context, members suggested that all communication with participants was personalised. They also advised including social desirability statements to, for example, communicate the view that taking part was a positive thing to do. In addition, they felt that participants would be more likely to act on research communications and activities if they had consistent branding and used clear, concise language. Although WhatsApp was recommended by the group for communicating with participants, the research team were concerned about data security issues (such as GDPR) associated with this method and did not pursue it further. The PPI group had divided opinions on whether participants should receive research updates via project newsletters: although some felt it might help participants to feel valued by the research team (n = 3), others felt it might be seen as junk mail (n = 2). Overall, the PPI group did agree however that newsletters would be acceptable if they were friendly sounding, brief, and interesting. While the group felt that sending Christmas greetings could be perceived as annoying by participants, they did feel that participants would feel valued if they received birthday greetings.

All of these ideas were advanced to Stage 3

#### Stage 3: Focus groups with the target audience.

In terms of communication, participants felt that style was as important as substance. They advised that all messages should address participants by name and that the sender should be a researcher’s name, not the study’s name (e.g., ‘The Wrapped project team’). They also advised that messages should have a continuous look, not be overly scripted, and use attention grabbing subject lines for emails. Social desirability statements were considered useful, but participants cautioned that they must sound genuine. Participants also advised that any instructions should be clear and easy to understand.

Participants considered strategies that they thought would be useful for maintaining long term engagement and preventing attrition. They agreed that telling participants about the impact they were having on the research, such as through short project updates or newsletters, would be a good way to show them that they were valued and could potentially increase engagement. Opinions were mixed however on the utility of digital birthday greetings: some thought this was a nice gesture, others thought it was weird and might make participants feel uncomfortable. See S8 Table for more details, including participant quotes.

The following strategies were found to have mutual agreement at Stages 2 and 3 and were therefore included in the final recruitment and retention strategy and were not voted on at Stage 4:

- All participant communications should:
  - Have a clear purpose.
  - Address participants by name.
  - Be concise, using clear, simple language, and contain no jargon.
  - Contain motivating social desirability statements that do not induce participants to feel guilty.
- Newsletters to be sent quarterly

The remaining strategies were advanced to Stage 4

#### Stage 4: PPI workshop 3.

Below are the final set of strategies for engaging participants with the research, finalised by the PPI group in collaboration with the research team. For more details, including the final votes and PPI quotes, see S11.

- Communications should be sent at 5:00pm as this is the most opportune time for participants to respond immediately; communications can be sent on any day of the week.
- Email should be the main method of communication.
- Messages should be from a researcher’s name but should not include pictures of the research team: given the sensitive nature of sexual health it was felt that including pictures would make participants feel less anonymous.
- Messages should be current and fun, while also professional in tone, but not make people feel uncomfortable; they should also refer to current events, such as holiday periods (to give a less automated feel).
- If possible, data collection should be avoided over the Christmas holiday period, as participants would likely be more preoccupied than usual, which could lead to lower response rates.
- Newsletters should be sent by email, should be an individual feature in their own right (i.e., not combined with any other communications such as reminders), and if possible, should include an opt-out option.
- Birthday greetings should not be sent- too much potential for participants to feel uncomfortable.

#### Stage 5: Observation of strategies during fRCT.

One minor amendment was made to engaging participants with research strategies. All data collection took place via REDCap [21,22], a secure data capture and management platform. Due to limitations with REDCap functionality, it was not possible to tailor messages to be sent at 5:00pm, to reference seasonal events (such as holiday periods), or to withhold survey invitations over the Christmas and New Year period. The remaining strategies remained unchanged.

#### Stage 6: PPI workshop 4.

The PPI group recognised the challenges in being unable to tailor the content of the messages or to alter their timing; these changes could not be prevented and were considered acceptable so long as participants did not receive communications at unsocial-able hours.

### Nature of survey invitations

#### Stage 1: Rapid evidence review.

Seventeen studies described using multiple modes to invite participants to complete surveys including email, SMS (text message), or letters through the post (with paper-based survey enclosed) [29–35,43–47,50–62]. A few studies described sending a prompt (i.e., advance notification of forthcoming invites) a few days before their survey was scheduled to arrive [30–32,44,51–53]. These themes were carried through to Stage 2.

#### Stage 2: PPI workshops 1 and 2.

SMS for survey delivery was the leading choice from the PPI group, with the recommendation that combining SMS and email would most likely be successful in reaching all participants regardless of their communication preferences. Prompts sent three days prior to a research activity were thought to be acceptable provided they were brief in nature. All of these ideas were advanced to Stage 3.

#### Stage 3: Focus groups with the target audience.

Email was considered by participants to be the best method for sending survey invitations, whereas SMS was advised for prompts. To ensure that messages were acted on and to maximise retention, participants also felt that these messages should: be kept to a minimum to prevent feelings of bombardment, contain a call to action, and mention the financial incentive. SMS prompts were considered most suitable for alerting participants about more demanding and/or sensitive measures; in the context of the Wrapped fRCT, the prompts were suggested for reminding participants to check the post for their STI self-sampling test kit and to remind them it would arrive in discreet packaging. The use of prompts as advance notification of surveys was considered suitable by participants in the first two focus groups, who thought they could be useful for jogging memory, but participants in the final focus group felt they lacked an essential call to action and could be counterproductive (it could lead to participants switching off or ignoring communications if they associated them with not needing to do anything as a result of the email). See S8 for more details, including participant quotes.

The following strategy was found to have mutual agreement at Stages 2 and 3 and were therefore included in the final recruitment and retention strategy and were not voted on at Stage 4:

- SMS to be used for sending prompts.

The remaining strategies were advanced to Stage 4.

#### Stage 4: PPI workshop 3.

Below are the final set of strategies for nature of survey invitations, finalised by the PPI group in collaboration with the research team. For more details, including the final votes and PPI quotes, see S11.

- Email should be used for survey invitations.
- Prompts are to be used only for more demanding and/or sensitive data collection measures; in the context of the Wrapped fRCT this applied to the test kits: this communication can also be used to confirm participants’ postal address and provide reassurance that the kit will be posted in discreet packaging. Although the PPI group held mixed opinions on the utility of prompts for surveys and test kits, the research team decided to use only for test kits.

#### Stage 5: Observation of strategies during fRCT.

No changes were made to the strategies for nature of survey invitations.

#### Stage 6: PPI workshop 4.

No changes were made to the strategies for nature of survey invitations.

### Value and implementation of financial incentives

#### Stage 1: Rapid evidence review.

Fifteen studies referred to use of financial incentives as a strategy to encourage recruitment and maintain retention [29–42,44,50–55,57,58,61–63]. Most studies described providing the financial reward when a research activity was completed, but a few also described offering a small incentive upfront to further encourage completion (referred to as ‘Something for Nothing’). Three studies offered financial incentives that increased in value over time (‘Increasing Amounts’). Prize draws were used by seven studies. A single study offered a higher value incentive if participants completed the research activity on time, but if the researcher had to call the participant to collect their data over the phone, they received a lower value incentive. These themes were carried through to Stage 2.

#### Stage 2: PPI workshops 1 and 2.

Amazon vouchers were chosen by the research team for the financial incentives; this decision was made as they are easy to distribute digitally and easily redeemed by participants. The PPI group reviewed the various strategies for implementing financial incentives on completion of the first Padlet. The group favoured increasing amounts which was thought to be a good strategy for encouraging retention. ‘Something for Nothing’ was considered unacceptable as they felt vouchers should only be received for completing an activity. Via the second Padlet, members voted overwhelmingly for schedules offering increasing amounts. On the basis of this, the research team drafted two voucher schedules offering increasing amounts, with an example ‘Something for Nothing’ schedule, to show the focus group participants at Stage 3. Although considered unacceptable, ‘Something for Nothing’ was advanced to Stage 3 to give focus group participants the opportunity to comment on this.

#### Stage 3: Focus groups with the target audience.

Financial incentives in the form of vouchers were considered an essential element to successfully retain participants in the trial. Focus group participants favoured offering increasing amounts of vouchers as the trial progressed but were unable to agree on an exact voucher distribution schedule; this was advanced to Stage 4. As participants agreed with the PPI group that the schedule offering ‘Something for Nothing’ should not be used this was discarded. See S8 for more details including participant quotes.

#### Stage 4: PPI workshop 3.

Below is the final voucher schedule for value and implementation of financial incentives, finalised by the PPI group in collaboration with the research team. For more details, including the final votes and PPI quotes, see S11.

- Vouchers should be higher for completion of more demanding data collection measures (i.e., STI self-sampling kits) and be distributed in increasing amounts over the duration of the trial in the following manner:
  - Month 0 (joining the study and baseline survey): £5
  - Month 3 survey: £5
  - Month 3 test kit: £10
  - Month 6 survey: £10
  - Month 12 survey: £15
  - Month 12 test kit: £20

#### Stage 5: Observation of strategies during fRCT.

In a further effort to address lower rates of recruitment, a minor amendment to the voucher schedule was made to increase both the amount of research activities incentivised (reporting Month 0 STI test result and visiting either the intervention or control website was added to the schedule) and in the value of vouchers distributed; two increases were made, first to a total of £85 and then to £100.

#### Stage 6: PPI workshop 4.

PPI members found this change acceptable and were of the belief that higher amounts of financial incentives would likely result in higher recruitment rates but advocated that the lowest effective amount should be used.

### Reminders to complete research activities

#### Stage 1: Rapid evidence review.

Eighteen studies described sending reminders to complete research activities via email, SMS, telephone call, or a letter sent via post [29–55,58–62,64]. The total number of reminders sent ranged between one to six, as described by 15 studies (three studies did not indicate amount sent). Reminders were sent at different intervals, ranging from between three to seven days after the initial invite, as described by six studies (12 studies did not indicate the frequency of reminders sent). These themes were carried through to Stage 2.

#### Stage 2: PPI workshops 1 and 2.

The PPI group felt that the use of reminders was an acceptable strategy and likely to encourage the completion of research activities. They advised that reminders should always be friendly in nature, brief, and remind participants of the financial incentives and how their individual input makes a difference. The group felt that reminders should be sent by SMS, although other communication methods, such as email and telephone calls, were also considered acceptable for non-responsive participants. The group also advised using an increasing scale of reminders, depending on the amount of effort required to complete the activity. For the fRCT, participants were required to complete surveys and STI self-sampling kits. As the kits required taking a biological sample, completing enclosed paperwork, and posting the kit back to laboratory, the group felt that this warranted additional reminders than for online survey completion. All of these ideas were advanced to Stage 3.

#### Stage 3: Focus groups with the target audience.

Participants felt that reminders should be sent by SMS, be friendly in tone, and emphasise the financial incentives of completion. They further advised that a completion deadline should be included to motivate completion. See S8 for more details, including participant quotes.

The following strategies were found to have mutual agreement at Stages 2 and 3 and were therefore included in the final recruitment and retention strategy and were not voted on at Stage 4:

- Reminders should be sent via SMS.

The remaining strategies were advanced to Stage 4.

#### Stage 4: PPI workshop 3.

Below are the final set of strategies for reminders to complete research activities, finalised by the PPI group in collaboration with the research team. For more details, including the final votes and PPI quotes, see S11 Table.

- A maximum of three reminders should be sent for each data collection measure.
- Reminders to be sent at different intervals according to the effort and time required for completion; shorter intervals for activities requiring less effort and time. For example, the Wrapped fRCT surveys were perceived as requiring less effort and time to complete as compared to the test kits.
- Reminders for surveys to be sent five days apart.
- The first test kit reminder should be sent three days after the participant receives their test kit, with the remainder sent 10 days apart. Although the PPI group indicated that all test kit reminders should be sent 10 days apart, the research team felt that an early first reminder would be a good opportunity to nudge completion.
- Reminders should highlight the financial incentives.

#### Stage 5: Observation of strategies during fRCT.

No changes were made to the strategies for reminders to complete research activities.

#### Stage 6: PPI workshop 4.

No changes were made to the strategies for reminders to complete research activities.

### Actions to minimise non-response

#### Stage 1: Rapid evidence review.

Five studies described approaches used to collect data should a participant not complete a planned research activity (e.g., survey) following any reminders being sent [29,44,46–49,51–53]. These included:

- Asking participants to complete a reduced set of primary outcome measures.
- Calling participants to either complete the survey with the researcher over the phone or offering support to enable the participant to complete themselves online.
- Sending the materials for a second time by post.

These themes were carried through to Stage 2

#### Stage 2: PPI workshops 1 and 2.

Telephone calls to non-responders was considered acceptable by the PPI group who felt that these could increase retention. They also felt that it was acceptable to use these to ask a limited number of questions from the relevant survey. They advised that the nature of the telephone calls should be focussed on checking on the wellbeing of participants; they felt that unexpected phone calls that focussed solely on data collection could lead to participant discomfort or irritation. All of these ideas were advanced to Stage 3.

#### Stage 3: Focus groups with target audience.

Participants advised that those who do not respond to data collection invitations be sent a personal email which has the tone of checking-in to make sure that everything is alright, rather than making them feel guilty for not completing in the first place. They also advised reminding participants about the financial incentive at this point. Participants felt that non-responders should not be contacted by telephone. This was felt too intrusive. A letter asking for confirmation of email and phone number instead was considered more acceptable. See S8 for more details, including participant quotes.

All of the strategies for actions to minimise non-response were found to have mutual agreement at Stages 2 and 3 and were therefore included in the final recruitment and retention strategy and were not voted on at Stage 4:

- Non-responsive participants should be sent a friendly email that clearly states the outstanding research activity and how to complete it, emphasizing the financial incentives.
- Phone calls to non-responsive participants should not be made as this could feel too intrusive. A letter should be posted to check if the participant’s contact details are correct.

#### Stage 4: PPI workshop 3.

All strategies for actions to minimise non-response were agreed at the conclusion of Stage 3 and were therefore not discussed further at Stage 4.

#### Stage 5: Observation of strategies during fRCT.

Although the finalised strategies specified non-responders would not receive phone calls as these might be too intrusive and lead to attrition, brief discreet phone calls were made to non-responders to ensure they had received the survey or test kit and to help resolve any barriers they might have experienced. The hope was that this would increase retention.

#### Stage 6: PPI workshop 4.

Providing that phone calls were conducted discreetly and sensitively, PPI members agreed that calling a non-responsive participant to attempt to engage them was appropriate and acceptable.

## Discussion

The aim of this paper is to describe the systematic process to develop a recruitment and retention strategy and offer an example of how this process can be replicated. A six-stage iterative process was followed, where a rapid evidence review informed an iterative consultation with a PPI group, focus groups with the target population, and observation of how the strategy functioned in a fRCT. The strategy includes a set of adverts (highlighting different value propositions); a schedule of financial incentives (per activity, increasing amounts); instructions to send one survey invite by email, one STI self-sample prompt by SMS, and up to three SMS reminders for both; plans for how to keep participants engaged (e.g., newsletters, personalisation of all communications); and information on the procedure to follow if participants fail to complete a research activity following all reminders (follow-up email/phone call). The fRCT recruited 230 participants as planned and exceeded the 60% retention target (at the 12-month endpoint); see Newby et al for full findings [65]. A definitive RCT is now planned. Although it cannot be said with confidence that the development of the strategies directly contributed to the successful fRCT recruitment and retention, it does add to the body of evidence about what works to optimise recruitment and retention, particularly in the context of internet-delivered trials involving young people and sensitive data. Although some of the specific strategies may only be applicable to online trials (i.e., sending surveys by email), all trials can benefit from the process followed to develop a comprehensive recruitment and retention strategy. Researchers may like to follow the process outlined in this paper in the development of their own recruitment and retention strategy.

Evidence concerning which strategies work best to increase trial participation is limited. This was the driving factor behind us undertaking our own work to identify what might work best for our trial. However, while we have provided a roadmap here for others wishing to develop their own strategy, and some tentative suggestions of what might work, we are not in a position to recommend any strategies with confidence. To do so requires undertaking randomised studies in which different types of strategy are compared and the effect on recruitment and/or retention measured. A meta-analysis combining the evidence from such studies has been undertaken [2] and while informative, notable weaknesses in the evidence base are identified, including many of the studies being small and almost half involving hypothetical scenarios [2].

While development of our recruitment and retention strategy was labour intensive, we felt that this was a good investment in time and yielded returns in the form of high levels of participation, smooth trial conduct for researchers, and a positive experience for participants. Once the plan was in place for how the trial would proceed, this was put into operation and very few changes were made during delivery. The final strategy was flexible enough to accommodate changes in response to unexpected issues, though adaptations made during the trial were in fact minimal. Positive feedback was also received from participants about their experience, which we believe is testament to the effort we put into getting things right for them from the start. This feedback, collected in our final follow-up survey, included for example: *“It was nice and straightforward to complete”* and *“You guys are always so lovely and so friendly. So knowledgeable about everything you’re doing as well. I’ve loved being a part of this project,”* with multiple participants indicating they enjoyed taking part. That participants felt valued by the team was particularly heartening for us to hear, as considerable effort was put into achieving this, for example through regularly thanking participants for their continued input into the research. Notably, feeling valued has previously been identified as essential to the participant experience [66].

This six-stage process benefitted from substantial input from a PPI group. Involvement of the group in the development of the strategy was considered by the research team to have been integral to the success of the fRCT in meeting its recruitment and retention targets. The benefit of PPI in optimising recruitment has been demonstrated in previous research. A meta-analysis of 19 clinical trials found that PPI input increased the odds of participant recruitment by 16%; the benefit to retention (assessed across five studies) was equivocal due to the paucity of eligible studies [67]. PPI also adheres to the Clinical Trials Transformation Initiative’s advice to engage with key stakeholders, such as PPI groups, in the development of recruitment and retention strategies [18]. Input from the PPI group identified strategies that were likely to be acceptable to the target sample, such as sending newsletters, but also importantly, those that were likely to be unacceptable and could potentially lead to participant disengagement, such as sending birthday wishes. Effectively engaging PPI groups offers researchers a unique perspective that can highlight potential barriers to participation and engagement in research [68].

Barriers to the participation of diverse PPI groups, that ideally represent the demography and relevant lived experience of the condition under examination, have also been identified [69]. The most notable limitation of the present research is the almost entirely female focus groups and the lack of diversity of the PPI group. While all PPI members had lived experience of STI testing, having been recruited through a STI self-sampling service, they were also all White and within a narrow age range (20–24 years). Furthermore, their sexual identity and experience of deprivation was unknown. Groups most at risk of STIs include those who are young (aged 15–24 years), Black, live in the most deprived areas, and are Gay, Bisexual or other men who have sex with men (GBMSM) [70]. In the fRCT, there was tentative evidence of lower recruitment and/or retention of participants from some of these at-risk groups (for further details see Newby et al [65]). The reasons for this are unknown and warrant further exploration. It may be that aspects of the recruitment and retention strategy did not account for the differing needs of this diverse population, especially given the sensitive nature of the data being collected. This will be addressed in our future planned RCT through recruiting a PPI group that has good representation from all at-risk groups and by tasking them to review the strategy from an inclusivity perspective. A further limitation was size of the PPI group; only six members in total. This did not provide sufficient depth in numbers to accommodate the lack of availability of some members at planned meetings or drop-out experienced across the three-year project. Although five members contributed to PPI workshops one and two, only three contributed to workshops three and four when the strategy was finalised for the fRCT and then subsequently reviewed in preparation for the definitive trial.

The research team also reflected on the process followed to develop the recruitment and retention strategy at study end. This led to the conclusion that the process could potentially be consolidated. In Stage 2, a series of two workshops were held with the PPI group: the first of which was to build on the ideas from the Stage 1 rapid evidence review, and a second of which was for members to vote on the best strategies to take forward to Stage 3 focus groups with target sample. A more targeted approach could be taken to consolidate these workshops, where members complete the Padlet in advance of a meeting at which more nuanced discussion and decision making takes place. The second workshop could then be removed. It was agreed however that all other stages/activities were required.

Planning for successful trials needs to incorporate planning for successful recruitment and retention. The six-stage process outlined in this paper can be applied to support the development of a recruitment and retention strategy for any other type of research. Researchers are advised to follow this, adjusting the parameters of the rapid evidence review to ensure that this aligns with design of the proposed research. The finalised strategy presented here may be applicable to other contexts, but consultation with the target participant group is still advised to ensure that the identified strategies can be effectively applied and are acceptable. Creating a comprehensive recruitment and retention strategy prior to trial commencement may lead to decreased researcher and participant burden and ultimately maximise the size and representativeness of the sample available for analysis, producing more robust results. Further research is needed to confirm if developing and implementing recruitment and retention strategies as described leads to increased recruitment and retention rates at the full trial stage.

## Conclusion

This paper presents a novel process for researcher and PPI co-development of a recruitment and retention strategy that incorporates rapid evidence review and empirical data collection. The strategies identified may be helpful to other researchers wishing to maximise recruitment and retention in their study. Equally, to ensure relevance to a particular research context and target sample, the process outlined in detail in this paper can be replicated.

## Supporting information

S1 Table Focus Group demographics. (DOCX)

S2 Table Focus Group Schedule. (DOCX)

S3 Table Articles included in rapid evidence review. (DOCX)

S4 Table Value propositions identified in the rapid review (Stage 1). (DOCX)

S5 Table Stage 2 Value Proposition Development. (DOCX)

S6 Table Potential adverts created by focus group participants. (DOCX)

S7 Table Elements of value propositions considered most important by focus group participants (Stage 3). (DOCX)

S8 Table Focus group participant (Stage 3) feedback on provisional strategies. (DOCX)

S9 Table Adverts and PPI voting outcomes at stage 4. (DOCX)

S10 Table PPI voting on advert component parts (stage 4). (DOCX)

S11 Table Final PPI voting on strategies for use in fRCT. (DOCX)
