# Supplementary material for: Planning for successful participant recruitment and retention in trials of behavioural interventions: Feasibility randomised controlled trial of the Wrapped intervention
Source: PLOS Digit Health. 2025 May 29;4(5):e0000875. doi: 10.1371/journal.pdig.0000875 (PMC12121807; doi:10.1371/journal.pdig.0000875)
Supplement: S1 Table — (DOCX) [file pdig.0000875.s001.docx]

**S1. Demographic characteristics of focus group participants**

| **Characteristic** | **Undergraduate Psychology Students (n=8)** | **Young People Associated with Care System (n=2)** | **Youth Theatre Organisation (n=5)** | **Total (n=15)** |
| --- | --- | --- | --- | --- |
|  | **n** | **n** | **n** | **n** |
| **Age** (r**ange and mean)** | | | | |
|  | 19-22  (20.4) | 22  (22) | 18-22  (19.8) | 18-22  (20.4) |
| **Ethnicity (n)** | | | | |
| White English/Welsh /Scottish/Northern Irish/ British, Irish | 3 | 2 | 2 | 7 |
| Any other White background | 2 | 0 | 1 | 3 |
| African | 1 | 0 | 0 | 1 |
| Any other Black/African/Caribbean background | 1 | 0 | 0 | 1 |
| Pakistani | 1 | 0 | 0 | 1 |
| White and Black Caribbean | 0 | 0 | 1 | 1 |
| Any other Mixed/Multiple ethnic background | 0 | 0 | 1 | 1 |
| **Gender** (n) | | | | |
| Female | 8 | 1 | 4 | 13 |
| Male | 0 | 1 | 1 | 2 |
