# Supplementary material for: Planning for successful participant recruitment and retention in trials of behavioural interventions: Feasibility randomised controlled trial of the Wrapped intervention
Source: PLOS Digit Health. 2025 May 29;4(5):e0000875. doi: 10.1371/journal.pdig.0000875 (PMC12121807; doi:10.1371/journal.pdig.0000875)
Supplement: S2 Table — (DOCX) [file pdig.0000875.s002.docx]

**S2. Table Focus Group Schedule**

| **Category** | **Questions** |
| --- | --- |
| Value Propositions/adverts | We want people to see a short message on the freetest.me website that grabs their attention and makes them want to join the study there and then. On the flip side, we want to attract people who want to commit to the study for the full 12 months, not just 5 minutes. |
| Now You're in Our Study, Please Stay! | What can we as a research team do to support and engage participants to keep completing the surveys and chlamydia test kits? |
| Prompts: How Delivered? | Prompts have been used by other studies as a way to message people before they receive the survey or test kit to let them know it is coming and pretty please to complete it. |
| Prompts: How Far in Advance? | How Many Days Ahead of a Survey or Test Kit Should We Send a Prompt? |
| How Should We Send Out Surveys? | The surveys will be online. What is the best, most reliable, better guarantee someone will actually see that we've sent them the survey and then go on to complete it? |
| Reminders: How Delivered? | Reminders have been used by other studies to help encourage people who may have forgotten to complete the survey or test kit. How should we send them out to participants that will guarantee people will see them and take action to complete it? |
| Reminders: How Long After Survey or Test Kit? | How long after we've sent a survey or test kit should we send a reminder to someone who hasn't completed? |
| Reminders: How Many Total? | How many total reminders should we send to someone who hasn't completed? |
| Thinking about the prompts, surveys, and reminders, what is the best time of day to catch someone? | The hope is that participants will receive the survey and immediately act on it and fill it out. Or in the case of the reminders, they will remember to take action on either the survey or the test kit. |
| Voucher Strategies | Taking part in research is time consuming and we want to recognize that participants are freely giving up their time for us. Sending out vouchers to participants is a frequently used method to thank participants for their efforts. Other past studies have used different strategies to distribute vouchers. We'd like your honest thoughts on how successful they might be in encouraging people to complete the surveys and test kits and potentially how they might backfire. |
| Vouchers | The next big question is how to divide the vouchers up according to each research activity completed. |
| Long Term Engagement in the Study | A lot can happen in 12 months! Despite this we still want people to remember the study and continue to complete surveys and test kits when they arrive. For the first 6 months something happens in the study every 3 months, but then we go quiet for a whole 6 months. Is there anything we could do as a research team to let participants know we're still whirring away in the background so that when the 12-month survey and test kit come around people aren't wondering who the heck we are. What are your ideas about keeping people in the loop about the study in a useful, not annoying way? |
