# Supplementary material for: Planning for successful participant recruitment and retention in trials of behavioural interventions: Feasibility randomised controlled trial of the Wrapped intervention
Source: PLOS Digit Health. 2025 May 29;4(5):e0000875. doi: 10.1371/journal.pdig.0000875 (PMC12121807; doi:10.1371/journal.pdig.0000875)
Supplement: S3 Table — (DOCX) [file pdig.0000875.s003.docx]

**S3. Articles included in rapid review**

| **Title** | **Authors** | **Year** | **Study Design** | **Population** |
| --- | --- | --- | --- | --- |
| The sexunzipped trial: Optimizing the design of online randomized controlled trials | Bailey, Julia V; Pavlou, Meneloas; Copas, Andrew; et al | 2013 | fRCT | 16-20 year olds in UK |
| The Men's Safer Sex project: intervention development and feasibility randomised controlled trial of an interactive digital intervention to increase condom use in men. | Bailey, Julia V; Webster, Rosie; Hunter, Rachael; et al | 2016 | fRCT | 159 Men aged ≥ 16 years; high risk of future STI [i.e., two or more partners in the past year (male or female) and non-condom use in the past 3 months, or symptoms of suspected acute STI, or seeking treatment for a STI]; at least half of their sexual partners are female. |
| A web-based cognitive behavior therapy intervention to improve social and occupational functioning in adults with type 2 diabetes (the SpringboarD trial): Randomized controlled trial/A Web-Based Public Health Intervention to Reduce Functional Impairment and Depressive Symptoms in Adults With Type 2 Diabetes (The SpringboarD Trial): Randomized Controlled Trial Protocol/Recruiting to a randomized controlled trial of a web-based program for people with type 2 diabetes and depression: Lessons learned at the intersection of e-mental health and primary care | Clarke, J.; Sanatkar, S.; Baldwin, P.A.; et al/ Proudfoot, Judy; Clarke, Janine; Gunn, Jane, et al/Fletcher, S.; Clarke, J.; Sanatkar, S.; et al | 2019/2017/2019 | RCT | 723 adults with type 2 diabetes and mild to moderate depressive symptoms |
| Personalized digital interventions showed no impact on risky drinking in young adults: A pilot randomized controlled trial | Davies, E.L.; Lonsdale, A.J.; Hennelly, S.E.; et al | 2017 | RCT | 488 young people aged 18–30 who self-identified as a current drinker |
| Can text messages increase safer sex behaviours in young people? Intervention development and pilot randomised controlled trial. | Free C, McCarthy O, French RS, et al | 2016 | RCT | 200 16–24 years with a positive chlamydia test result or who had had unsafe sex in the last year |
| Improving adherence to smoking cessation treatment: Smoking outcomes in a web-based randomized trial/ Improving adherence to smoking cessation treatment: Smoking outcomes in a web-based randomized trial/ Improving adherence to web-based cessation programs: a randomized controlled trial study protocol | Graham, A.L.; Papandonatos, G.D.; Cha, S.; et al/Graham, A.L.; Papandonatos, G.D.; Cha, S.; et al./ Graham, Amanda L; Cha, Sarah; Papandonatos, George D; et al | 2017/2018/2013 | RCT | 5290 adult smokers |
| A Pilot Randomized Controlled Trial of a Digital Intervention Aimed at Improving Food Purchasing Behavior: The Front-of-Pack Food Labels Impact on Consumer Choice Study/Protocol for a pilot randomised controlled trial of an intervention to increase the use of traffic light food labelling in UK shoppers (the FLICC trial) | Harrington, R.A. Richard A; Scarborough, Peter; et al/Peter Scarborough, Peter; Hodgkins, Charo; Raats, Monique M; et al | 2019/2015 | RCT | 496 grocery store loyalty card shoppers |
| A Pilot RCT of an Internet Intervention to Reduce the Risk of Alcohol-Exposed Pregnancy | Ingersoll, K.; Frederick, C.; MacDonnell, K.; et al | 2018 | pilot RCT | 71 women with risky drinking behaviours and at risk of unintended pregnancy |
| Web-based integrated bipolar parenting intervention for parents with bipolar disorder: a randomised controlled pilot trial/An exploratory randomised controlled trial of a web-based integrated bipolar parenting intervention (IBPI) for bipolar parents of young children (aged 3–10) | Jones, S.H.; Jovanoska, J.; Calam, R.; et al/ Jones, Steven; Wainwright, Laura D; Jovanoska, Jelena; et al | 2017/2015 | fRCT | 97 parents with a diagnosis of bipolar disorder |
| Web-based intervention using behavioral activation and physical activity for adults with depression (the emotion study): Pilot randomized controlled trial | Lambert, J.D.; Greaves, C.J.; Farrand, P.; et al | 2018 | fRCT | 62 adults with moderate depressive symptoms |
| Complaint-directed mini-interventions for depressive complaints: A randomized controlled trial of unguided web-based self-help interventions | Lokman, S.; Leone, S.S.; Sommers-Spijkerman, M.; et al | 2017 | RCT | 329 adults with mild to moderate depressive symptoms |
| Effects on engagement and health literacy outcomes of web-based materials promoting physical activity in people with diabetes: An international randomized trial | Muller, I.; Rowsell, A.; Stuart, B.; et al | 2017 | RCT | 1045 adults with type 2 diabetes |
| Does the structure (tunneled vs. free-roam) and content (if-then plans vs. choosing strategies) of a brief online alcohol intervention effect engagement and effectiveness? A randomized controlled trial | Norman, P.; Webb, T.L.; Millings, A.; Pechey, L. | 2019 | RCT | 286 adults |
| Young adults' engagement with a self-monitoring app for vegetable intake and the impact of social media and gamification: Feasibility study | Nour, M.; Chen, J.; Allman-Farinelli, M. | 2019 | fRCT | 97 young adults |
| Guided web-based treatment program for reducing cannabis use: A randomized controlled trial | Sinadinovic, K.; Johansson, M.; Johansson, A.-S.; et al | 2020 | RCT | 303 cannabis users |
| A Digital Intervention Addressing Alcohol Use Problems (the "Daybreak" Program): Quasi-Experimental Randomized Controlled Trial. | Tait, Robert J; Paz Castro, Raquel; Kirkman, Jessica Jane Louise; et al | 2019 | RCT | 793 adult users of an easily accessed alcohol app on app store |
| Methodological and ethical challenges in a web-based randomized controlled trial of a domestic violence intervention/ An online healthy relationship tool and safety decision aid for women experiencing intimate partner violence (I-DECIDE): a randomised controlled trial/ Protocol for a randomised controlled trial of a web-based healthy relationship tool and safety decision aid for women experiencing domestic violence (I-DECIDE) | Tarzia, L.; Valpied, J.; Koziol-McLain, J.; et al/ Hegarty, Kelsey; Tarzia, Laura; Valpied, Jodie; et al/ Hegarty, Kelsey; Tarzia, Laura; Murray, Elizabeth; et al | 2017/2019/2015 | RCT | 422 women experiencing intimate partner violence or experiencing fear of intimate partner |
| Smoking cessation intervention trial outcomes for sexual and gender minority young adults./ Facebook recruitment of young adult smokers for a cessation trial: Methods, metrics, and lessons learned/ A randomized controlled evaluation of the tobacco status project, a Facebook intervention for young adults/ The Tobacco Status Project (TSP): Study protocol for a randomized controlled trial of a Facebook smoking cessation intervention for young adults | Vogel, Erin A; Thrul, Johannes; Humfleet, Gary L; et al/ Ramo, Danielle, E; Rodriguez, Theresa M.S.; Chavez, Kathryn; et al/ Ramo, Danielle, E; Thrul, Johannes; Delucchi, Kevin L.; et al/ Ramo, Danielle, E; Thrul, Johannes; Delucchi, Kevin L.; et al | 2019/2014/2018/2015 | RCT | 500 18-25 year old smokers |
| Pilot phase of an internet-based RCT of HIVST targeting MSM and transgender people in England and Wales: Advertising strategies and acceptability of the intervention | Witzel, T.C.; Gabriel, M.M.; McCabe, L.; et al | 2019 | RCT | 1035 men and transgender women who have sex with men |
| The Men's Safer Sex project: intervention development and feasibility randomised controlled trial of an interactive digital intervention to increase condom use in men. | Bailey, Julia V; Webster, Rosie; Hunter, Rachael; et al | 2016 | fRCT | 159 Men aged ≥ 16 years; high risk of future STI [i.e., two or more partners in the past year (male or female) and non-condom use in the past 3 months, or symptoms of suspected acute STI, or seeking treatment for a STI]; at least half of their sexual partners are female. |
| Relative effectiveness of a full versus reduced version of the ‘smoke free’ mobile application for smoking cessation: An exploratory randomised controlled trial [version 2; referees: 2 approved] | Crane, D.; Ubhi, H.K.; Brown, J.; West, R. | 2019 | RCT. Participants were not recruited: participants had downloaded the Smoke Free app and were informed their data was being used for research purposes, which they were asked to consent (unsure if users could use the app without consenting to research) | 28,112 Adult smokers |
| Extending access to a web-based mental health intervention: Who wants more, what happens to use over time, and is it helpful? Results of a concealed, randomized controlled extension study | Hensel, J.M.; Shaw, J.; Ivers, N.M.; et al | 2019 | RCT | 112 previous participants in trial for people seeking mental health treatment |
| OpenSIMPLe: A real-world implementation feasibility study of a smartphone-based psychoeducation programme for bipolar disorder | Hidalgo-Mazzei, D.; Reinares, M.; Mateu, A.; et al | 2018 | RCT | 201 adults with bipolar disorder receiving pharmacological treatment |
| Web-based self-management support for people with type 2 diabetes (HeLPDiabetes): Randomised controlled trial in English primary care/HeLP-Diabetes: randomised controlled trial protocol | Murray, E.; Sweeting, M.; Dack, C.; et al/ Murray, Elizabeth; Dack, Charlotte; Barnard, Maria; et al | 2017/2015 | RCT | 374 adults with type 2 diabetes |
| Effect of communicating phenotypic and genetic risk of coronary heart disease alongside web-based lifestyle advice: The INFORM Randomised Controlled Trial/Information and Risk Modification Trial (INFORM): design of a randomised controlled trial of communicating different types of information about coronary heart disease risk, alongside lifestyle advice, to achieve change in health-related behaviour | Silarova, B.; Sharp, S.; Usher-Smith, J.A.; et al./ Silarova, Barbora; Lucas, Joanne; Butterworth, Adam S.; et al | 2019/2015 | RCT | 956 blood donors with no history of CVD risk |
| Evaluation of a web-based intervention providing tailored advice for self-management of minor respiratory symptoms: Exploratory randomized controlled trial | Yardley, L.; Joseph, J.; Michie, S.; Weal, M.; et al | 2010 | pilot RCT | 714 university students with minor respiratory symptoms (coughs and colds) |
