# Supplementary material for: Planning for successful participant recruitment and retention in trials of behavioural interventions: Feasibility randomised controlled trial of the Wrapped intervention
Source: PLOS Digit Health. 2025 May 29;4(5):e0000875. doi: 10.1371/journal.pdig.0000875 (PMC12121807; doi:10.1371/journal.pdig.0000875)
Supplement: S4 Table — (DOCX) [file pdig.0000875.s004.docx]

**S4. Types of value propositions identified in the rapid evidence review (Stage 1)**

| **Strategy** | **Definition** | **Example Message** |
| --- | --- | --- |
| Altruism: help with research | Messages focused on how potential participants could contribute to science by joining the study. | “Interested in sexual health? Willing to help us with our research?” (29) |
| Altruism: potential to help others | Messages emphasised how potential participants would be helping improve things for people similar to themselves by joining the research. | No example message provided |
| Financial incentive | Messages directly mentioned that participants would earn money and/or vouchers through their involvement in the research. | “Looking for people who smoke. Join and you can get up to $180. Click here to learn more.” (40) |
| Improve health and/or wellbeing | Messages aimed to reach specific populations with targeted messages highlighting how the research might improve their health and/or wellbeing. | “Feeling tired? Essential nutrients in vegetables can enhance your wellbeing. Learn how to eat a little more veg every day through the 4-week smartphone program designed by researchers at The University of Sydney.” (34) |
| Scarcity of spaces in trial | Messages highlighted how only a few spaces were available for the research. | “Only a few spaces left in Tobacco Status Project. Click to see if you are eligible.” (40) |
| It’s easy to take part | Messages indicated that being a participant in the study would be easy to complete. | No example message provided |
| Messages were created in collaboration with experts/members of the target population | Messages were specifically created with user groups and subject experts, such as organisations that work directly with the target population. | No example message provided |
| Professional design/use of logos in order to indicate message coming from expert source | Recruitment messaging maintained a continuous look and tone so as to convey it was coming from a trusted source. | No example message provided |
