# Supplementary material for: Planning for successful participant recruitment and retention in trials of behavioural interventions: Feasibility randomised controlled trial of the Wrapped intervention
Source: PLOS Digit Health. 2025 May 29;4(5):e0000875. doi: 10.1371/journal.pdig.0000875 (PMC12121807; doi:10.1371/journal.pdig.0000875)
Supplement: S5 Table — (DOCX) [file pdig.0000875.s005.docx]

**S5. Table Stage 2 Value proposition development**

| **Value proposition category** | **Adverts drafted at workshop one** | **Adverts presented at workshop two** | **Votes at workshop two** | **Proceed to Stage 3?** |
| --- | --- | --- | --- | --- |
| Altruism | If you're getting tested 4 times a year already, how would you like to turn that into making a positive change for everyone's sexual health? Click here to and out more. | No change | 1 yes, 0 no, 4 omitted | Yes |
| Altruism | Looks like you're doing your bit to keep yourself and others safe, would you like to help us improve access to information about sexual health? | No change | 4 yes, 0 no, 1 omitted | Yes |
| Altruism | Interested in being a part of a movement towards better sexual health for young people? | No change | 0 yes, 0 no, 5 omitted | No |
| Altruism; Financial incentive | Are you interested in being paid to help develop safer health services? | No change | 0 yes, 0 no, 5 omitted | No |
| Improve health and/or wellbeing; Financial incentive | Want to subscribe to frequent testing kits? If so, get rewarded for your participation and feedback! | No change | 4 yes, 0 no, 1 omitted | Yes |
| Altruism; Financial incentive | Join our research team and get paid to improve sexual health services! | No change | 1 yes, 0 no, 4 omitted | Yes |
| Altruism; Financial incentive | Take part in a research study to improve sexual health services and receive high street vouchers for your time! | No change | 0 yes, 0 no, 5 omitted | No |
| Altruism; Financial incentive | Join a study to help improve Sexual Health services and get paid for your time | No change | 1 yes, 0 no, 4 omitted | Yes |
| Improve health and/or wellbeing |  | Sexual health is important for your wellbeing. Find out how you might improve your sexual health by joining our research. | 0 yes, 0 no, 5 omitted | No |
| Scarcity of spaces in trial |  | [Add "last few spaces" remaining to any of the value propositions] | 0 yes, 0 no, 5 omitted | No |
| Altruism; It’s easy to take part |  | Sexual health research is surprisingly easy to take part in, find out how you can make a difference today. | 0 yes, 0 no, 5 omitted | No |
| Altruism |  | You can make a difference to improve sexual health services, find out more about joining our research. | 4 yes, 0 no, 1 omitted | Yes |
