# Supplementary material for: Planning for successful participant recruitment and retention in trials of behavioural interventions: Feasibility randomised controlled trial of the Wrapped intervention
Source: PLOS Digit Health. 2025 May 29;4(5):e0000875. doi: 10.1371/journal.pdig.0000875 (PMC12121807; doi:10.1371/journal.pdig.0000875)
Supplement: S6 Table — (DOCX) [file pdig.0000875.s006.docx]

**S6. Table Potential adverts created by focus group participants**

| 1. ‘Join our study to help improve sexual health services whilst being paid!’ 2. ‘Less positive tests, more positive change’ |
| --- |
