# Supplementary material for: Planning for successful participant recruitment and retention in trials of behavioural interventions: Feasibility randomised controlled trial of the Wrapped intervention
Source: PLOS Digit Health. 2025 May 29;4(5):e0000875. doi: 10.1371/journal.pdig.0000875 (PMC12121807; doi:10.1371/journal.pdig.0000875)
Supplement: S7 Table — (DOCX) [file pdig.0000875.s007.docx]

**S7. Elements of value propositions considered most important by focus group participants (Stage 3)**

| **Element** | **Description** | **Quote** |
| --- | --- | --- |
| Brief, clear, and snappy | Messages need to be brief and to the point; each message should be able to be read and understood quickly. | *Participant 8: I also think this one's a bit wordier as well, and I think keeping it shorter will increase the chances of someone properly reading it as well.*    *Participant 5: You see, that one is quite good and snappy, to be fair. It's very snappy and down to the point.* |
| Mention financial incentive | Financial incentives were considered the most important element of a message as it would pique potential participants’ interest, encourage them to join the study, and take away some of the embarrassment of taking part in sexual health research. | *Participant 13: If there's an incentive at the end of it, I feel like people are more likely to be connected because they know that they're going to get something at the end of it, actually physical and they can use that little money.* |
| Altruism | Although not as strong a motivator as financial incentives, messages that draw attention to helping others and making a difference will encourage people to find out more about the study. | *Participant 14: Because it says you can make a difference, and I think that will resonate with people that want to help others.* |
| Convey that participation is valued | Messages should communicate that participants will be appreciated as an individual making a valued contribution and will not be treated as a research specimen. | *Participant 3: That sounds more interesting, I think, for a lot of people. Because it makes them sound like they're someone important, you know what I mean? People like to have the importance like on them, and it makes them sound like they're part of like a research team, which they kind of are, but they're obviously the participant.* |
| Indicate commitment required | To avoid significant dropout following baseline, messages should convey that an element of commitment will be required. | *Participant 7: say you can earn money by giving your ideas and your knowledge, and into what this is going to be. I'm not quite sure how you would want to word it, but, certainly, maybe have it earn money and you can earn this if you just put some effort in, put some work in. And not even that much, really, but you can still earn what we're going to give out and all we need is information from you.* |
| Feel easy to take part in | Participation needs to be described in a way that doesn’t feel difficult or time consuming. | *Participant 18: With that, “it looks like you're doing your bit message,” and the thing that I like about that is that it kind of creates like a low-pressure environment. Because, obviously, you want them to commit to the study, but it's kind of like you don't need to stress, we're not going to ask you to write a paper on it sort of thing. So I feel like that will entice people if you just kind of start off a bit casual, and then give a few details, and then once they click on it, you can explain more, if that makes sense?* |
