# Supplementary material for: Planning for successful participant recruitment and retention in trials of behavioural interventions: Feasibility randomised controlled trial of the Wrapped intervention
Source: PLOS Digit Health. 2025 May 29;4(5):e0000875. doi: 10.1371/journal.pdig.0000875 (PMC12121807; doi:10.1371/journal.pdig.0000875)
Supplement: S8 Table — (DOCX) [file pdig.0000875.s008.docx]

**S8. Table 3 Focus group participant (Stage 3) feedback on provisional strategies**

| **Retention Strategy** | **Description** | **Quote** |
| --- | --- | --- |
| **Engaging participants with the research** | | |
| Personalised communications with each participant | In all messaging, participants should be addressed by name and the sender should be a person’s name, not the name of the study. | *Participant 3: It's fair to speak to them by person, you know what I mean? It just shows more effort, doesn't it? So, yeah, it incites more effort on their behalf too, because someone's making an effort towards you, and you feel more inclined to make effort towards them.*  *Participant 18: Also - it's also if it's possible for the sender of the email, you know the thing that appears is this person has sent… That's the sender. Could you make the sender a person's name, instead of the name of like a company?* |
| Continuity of materials (logos, colours, fonts, templates, formatting) | Messages should have a continuous look, i.e., logos, colours, fonts, etc. | *Participant 9: Well, I think it's very important [having a continuous look], because I feel like you know it's all coming from the same place. I really know it's all - I don't know, you just tie it altogether in your head. I don't really know why else it's important, but I just think it's important to know that it's the same people that care about you as a human being; it's coming from them, and it's not coming from another place.* |
| Clear, simple, brief and impactful  instructions/communications | Message shouldn’t feel overly scripted, should feel fun and relevant.    Subject lines for emails should be attention grabbing and playful in nature.    Genuine and encouraging social desirability statements would be effective, but statements that sound false or make participants feel bad about themselves would backfire.  Instructions should be clear, easy to read, free of jargon, and with a series of steps with accompanying pictures if applicable. | *Participant 7: So I have joined this thing and it's called Uniboob, where they send messages every month just to check your bodies for cancerous lumps, or anything. But they don't go, hey, oh my God, you're going to die! They go, yo, it's like Halloween check your boobs, check your muscles, check your pecs and it's really fun.*  *Participant 13: Yeah, on the point of email marketing, yeah, like you were saying with the headings, because even add like a bit of humour into it to captivate people, and get them to click on the email, something like, oh hey, we're not just a one-night stand, we're in like a relationship.*  *Participant 7: Just making sure it [social desirability statement] doesn't sound sarcastic - you're doing really well! Literally, yeah, because I think it's nice to have a little message like, you know what? Thank you for completing this, this is really helping people. And then having that most people are doing this, so you're almost good enough. I don't know, like trying to convey the right meaning, like thank you for what they're doing.*  *Participant 8: I'd say probably brief, but really sort of clear, which is a hard balance to find, but you don't want a massive message with lots of tiny writing or something, because then it'll just seem really tiresome to read. But if there was something that was fairly simple with like numbers one, two, three, and maybe like images, then you've already got so much information just from a quick look, without having to read every single line to know what you're doing.* |
| Tell Participants the Impact They’re Having | Tell participants the difference they are making to the trial – this will make them feel valued, thereby increasing completion of data collection measures. | *inform them that they will have a direct/important impact on sexual health services ― ANONYMOUS [Psych Undergrads Padlet]*  *make it clear the exact impact they will make, i.e. make an impact on the number of other young people who put themselves at risk from catching chlamydia ― ANONYMOUS [Psych Undergrads Padlet]*  *Maybe explain some key stats about sexual health (e.g. chlamydia is present in 1 in X amount of people) so they feel that it is an important thing to help improve ― ANONYMOUS [Psych Undergrads Padlet]* |
| Send project Updates or Newsletters | Newsletters would be a good method of showing participants the impact they were having and were considered effective for maintaining engagement throughout the trial. | *Participant 3: if you could give them like some results or something, or like say that you give them some results of the research, then, obviously, that would keep them more interested, because then they can see the direct impact they've had.* |
| Send birthday Greetings | Mixed opinions on the role of birthday greetings: some felt it would be a kind gesture that would in turn encourage continued participation, others though it was weird and awkward. | *Participant 19: That sounds good. That sounds really good. Yeah, it does, it makes the person feel special, so then the whole thing around teamwork and how you're part of this, and you're part of this doesn't just sound like what is being said. But, oh my God, they actually remember my birthday, yeah.*  *Participant 3: I think that's weird! I don't even send half of my friends one, so why would I get one off of them? I don't know, I think that's really weird, getting a card off someone you don't know… it would be kind of weird I'd imagine, and it's like happy birthday from your sexual health clinic, or whatever it is.* |
| Nature of survey invitations | | |
| Survey invites by email | Surveys should be sent by email and should mention the financial incentive and how long it will take to complete. | *Participant 20: Emails should have the link [to the survey]. I think a good thing to like include in it, is how long this survey is going to take you, because if… I don't know, if I open it and I'm just about to go out the door, and I'm like it's going to take me five minutes, I might as well do it while I'm waiting for someone to get ready, or if I'm at work and I'm trying to pass some time, you might as well… I don't know, if it's only like five or ten minutes, but if it's going to take you an hour, I need to set time apart for it, because I don't have an hour of time right now.*  *Participant 13: Saying like payday is pending just to remind them that they are getting paid to do these surveys.* |
| Use of prompts | Prompts by text message for more demanding and/or sensitive activities (i.e. test kits) were perceived as valuable as it was a call to action to expect its arrival in the post in plain packaging and remind participants of the financial incentive.  Mixed opinions of the utility and effectiveness of prompts preceding the arrival of surveys. The first two focus groups thought it would be useful to jog their memory, but the final focus group perceived prompts as bombardment and would encourage non-responsiveness as it was not a call to action. | *Participant 12: But with the test kits, I think a prompt might be helpful for that, just that they are expecting it to come. Because I don't know what shape they come in or whatever, but whether it can fit through the letterbox…*  *Participant 18: So then, yeah, on the day itself, as well as a few days before, I think that will be nice, or a few days before so you get your mind ready, oh, this is going to happen. And on day, just so that in case you missed it and to remind you that, well, you're getting money for this, so you may as well do it.*  *Participant 14: But, personally, I would be more inclined to get a text or an email saying, oh, our survey is here and I just do it there and then. Whereas if I got a text or an email saying it's in a few days, I'm probably… It'll be on my mind, which is obviously a good thing, but then when I get the next email I might not be inclined to do the survey straightaway if I'm used to reading emails, and not acting upon them at the time.* |
| **Value and implementation of incentives** | | |
| Award increasing amount over time-period of trial; do not offer ‘something for nothing’ | While increasing amounts was considered acceptable and likely to provide motivation for participants to continue participating for the duration of the trial, something for nothing was mostly considered a terrible idea that would lead to low commitment and poor-quality data.  No consensus was reached on how to distribute the vouchers, although there was a preference for increasing amounts with the largest payment at the end of the trial | *Participant 18: Yeah, that is definitely a nice incentive knowing that. Not only am I getting paid for it, but I'm going to be getting more, so then I'll be looking forward to it. It's like, oh yeah, I get to get more money the next time, or the next time, I may as well just do everything.*  *Participant 3: Not with something for nothing. You're going to get people with no commitments.*  *Participant 7: Or certainly they continue on with the tests and stuff, they do it to a lower level of completeness, and you just click all the 'yes' answers, all the no answers and then they've gone. And that'll swerve any information that the study gets.*    *like that the last month has the most incentive so people actually finish it ― ANONYMOUS [Psych Undergrads Padlet]* |
| **Reminders to complete research activities** | | |
| Repeated (limited) reminders by SMS text message | Reminders were viewed positively for increasing response rates. Reminders should be sent by text message, feel casual in tone, and mention the financial incentive. Some focus group participants advocated for a completion deadline to encourage prompt participation. | *Participant 18: In the reminder about doing it, could there be something about how, well, that amount of whatever you're being paid is slipping out of your hands in just five minutes, it could be yours, just do this. You know you want to do it! Or just something like that, so that you are reminded that, well, you could potentially be getting money for it and that you'd be losing. I mean, I know myself, and I know that if I had the chance to make money and it only takes us five minutes to make that money, I wouldn't leave it on.*  *Participant 12: Sorry. I think with what someone said earlier, the idea of deadlines is quite good, because they are getting paid for it, so they should be providing you with what they committed to. But maybe like make them 24 hours or 48 hours, just so that they don't feel under pressure like, oh, if you don't do it in the next two hours you're not getting anything out of this. So maybe just like set loose deadlines, just to know that it needs to get done soon.* |
| **Actions to minimise non-response** | | |
| Tone of communication should be supportive | Non responders should be emailed, letting them know the researchers noticed they hadn’t completed a few measures, and ask if everything was alright. Messages should feel personal in tone, encouraging the participants to continue with a reminder of the financial incentives; messages should not make them feel guilty for having not completed something. | *Participant 18: If someone has not really done what they had to do. So the missing you, or it looks like you haven't done this, is there anything we should know about, or are you okay? Yeah, are you… Yeah, so like are you okay with something that is personal.*    *Participant 3: You could carrot and stick them with that. You could be like, oh, you can still get this money for doing this, and… Or you could be like, oh, your research is almost done, or you've already done this and you're almost done and you can get your money if you finish it.* |
| Do not use phone calls but letters ok | Non responders should never be called by telephone nor asked to complete a shortened version of the survey (key measures only). It would be acceptable to post a letter asking to confirm if their email address and phone number had changed, as this was a likely occurrence among young people. | *Participant 20: I think the only issue with like calling people now, is I think - at least, personally, a lot of people I know in the age range you're going to - they don't like being called; they're like very rarely on the phone. So I think you might then make them feel like they're being quite bombarded with this, and it might make them want to just disconnect from the study completely. So I think maybe trying to stay over email and text might be more useful, because of your age range.*  *Participant 3: I would worry a bit, because 16 to 24-year-olds, in that six months, the amount of people who would change their phone number is quite a bit. Like it won't be anywhere near all of them, but I'd say at least like 8 to 10 per cent of people will change their phone number in that time.*  *Participant 5: Yeah, that's what I was about to say, send a letter and just- do you know like that one you have to send, them little things with the free post envelopes, so they can send it back. Do one of them, and they can just, it can be their choice if they want to tick it or not, and send it back to you.* |
