# Supplementary material for: Planning for successful participant recruitment and retention in trials of behavioural interventions: Feasibility randomised controlled trial of the Wrapped intervention
Source: PLOS Digit Health. 2025 May 29;4(5):e0000875. doi: 10.1371/journal.pdig.0000875 (PMC12121807; doi:10.1371/journal.pdig.0000875)
Supplement: S9 Table — (DOCX) [file pdig.0000875.s009.docx]

**S9. Table Adverts and PPI voting outcomes at stage 4**

| **Advert** | **Voting** | **Quotes** |
| --- | --- | --- |
| Get paid to join a study that supports young people’s condom use. [included from Stage 3] | No votes | N/A |
| Join our research team and get paid to improve young people's sexual health. [included from Stage 3] | 3 yes, 0 no | N/A |
| You can make a difference to improve the sexual health of young people, find out more about joining our research. [included from Stage 3] | No votes | N/A |
| Want to subscribe to frequent testing kits? If so, get rewarded for your participation and feedback! [included from Stage 3] | 1 yes, 0 no | N/A |
| Looks like you're doing your bit to keep yourself and others safe, would you like to help us improve young people’s condom use by taking part in a study? [included from Stage 3] | No votes | N/A |
| Earn up to £65 in vouchers by taking part in a study to improve sexual health, find out how you can make a difference today. [created based on analysis of Stage 3] | 3 yes, 0 no | *This is my favourite choice* |
| Make a difference to improve sexual health  for young people- join our study and get paid for your time. [created based on analysis of Stage 3] | 2 yes, 0 no | N/A |
| Ready to help change young people’s sexual health for the better? Your input can make a difference: join our study and get paid for your time. [created based on analysis of Stage 3] | 2 yes, 0 no | N/A |
| Be a part of more positive change and less positive tests: make a difference by joining our study and get paid for your time. [created by focus group participants at Stage 3] | 3 yes, 0 no | *I really like the first part of this message; it's good fun and attention-grabbing. I can't stop my inner pedant from pointing out that it should be "fewer positive tests" though :D* |
| Your views are important: join our study to help improve sexual health for young people whilst being paid! [created by focus group participants at Stage 3] | 2 yes, 0 no | N/A |
