# Supplementary material for: Planning for successful participant recruitment and retention in trials of behavioural interventions: Feasibility randomised controlled trial of the Wrapped intervention
Source: PLOS Digit Health. 2025 May 29;4(5):e0000875. doi: 10.1371/journal.pdig.0000875 (PMC12121807; doi:10.1371/journal.pdig.0000875)
Supplement: S10 Table — (DOCX) [file pdig.0000875.s010.docx]

**S10. Table PPI voting on advert component parts (stage 4)**

| **Advert Component** | **Voting** | **Quotes** |
| --- | --- | --- |
| **Call to action** | | |
| Want to make a positive change? | No votes | N/A |
| We need your help! | 3 yes, 0 no | N/A |
| Views of young people needed! | No votes | N/A |
| Want to make a difference? | 3 yes, 0 no | N/A |
| Join our research team! | No votes | *Someone pointed out in the focus group that this may sound too*  *much like a full-time job* |
| **Description of the research** | | |
| Take part in a study to improve young people’s sexual health. | 3 yes, 0 no | N/A |
| Take part in a study on young people’s sexual health | No votes | N/A |
| Take part in a study on supporting young people’s condom use. | No votes | N/A |
| Support other young people to improve their sexual health by taking part in our study. | 3 yes, 0 no | N/A |
| **Incentive** | | |
| Get paid for your time. | 3 yes, 0 no | N/A |
| Get rewarded for your time. | No votes | *This is a bit more vague compared to the others* |
| Earn up to £65 in vouchers for your time. | 2 yes, 0 no | N/A |
| Last few spaces remaining! | No votes | N/A |
