# Supplementary material for: Planning for successful participant recruitment and retention in trials of behavioural interventions: Feasibility randomised controlled trial of the Wrapped intervention
Source: PLOS Digit Health. 2025 May 29;4(5):e0000875. doi: 10.1371/journal.pdig.0000875 (PMC12121807; doi:10.1371/journal.pdig.0000875)
Supplement: S11 Table — (DOCX) [file pdig.0000875.s011.docx]

**S11. Table Final PPI voting on strategies for use in fRCT**

| **All Messages Should:** | **Feedback** | **Votes** | **Quotes** |
| --- | --- | --- | --- |
| **Engaging participants with the research** | | | |
| Messages should be fun and relevant, especially subject lines for emails | There was agreement that messages should be fun but there was concern that the type of messages as suggested by the focus groups might make people feel uncomfortable. Important that have professional feel. | 2 yes, 1 no | *I like the idea of being fun, but I don’t like the vibe of the messages in the examples.. could just be me though! Maybe something along the lines of “Spent that voucher yet? We have another one waiting for you!”. Not really that fun I guess, but think it’s a catchy way to entice someone.* |
| Feel current- like mentioning seasonal holidays or events | Voting unanimously indicated this was acceptable. | 3 yes, 0 no | N/A |
| The sender should be a researcher’s name | Voting unanimously indicated this was acceptable. | 3 yes, 0 no | *I think this a really nice idea, or 'The Wrapped Team' rather than 'Project' maybe?* |
| Messages should contain photos of the research team | Including pictures of the research team was not considered a good idea as it may make participants feel uncomfortable. | 0 yes, 2 no | *Don’t think that’s too relevant, I guess it’s always nice to put a face to a name but not a necessity in this case. I think by  keeping things a little more anonymous participants would and it easier to be more open when answering questions.* |
| All messages should be sent midweek: Tuesday, Wednesday, or Thursday | Voting unanimously indicated that messages should be sent any day of the week as this would make no difference to response rates. | 0 yes, 3 no | N/A |
| Sent at a particular time of day (such as 12:00 or 5:00) | Voting unanimously indicated that 5:00 was an ideal time for all messages to be sent out as this would catch the majority of participants at an opportune time to respond immediately. | 3 yes, 0 no | *This way it will be fresh in participants minds when they get home and have time to complete activities* |
| If messages could only be email or SMS, but not both, what is the preference? | Emails were seen as preferable if only one communication method could be used, as it would be more accessible by multiple devices, would likely be easier for the research team to format the messages, and would be easier to personalise for participants, which would be better for retention. | 3 yes, 0 no | *Emails are available on both phones and computers, which might increase likelihood of participants seeing the messages. Formatting is also a bit easier/more customise-able* |
| Should we collect data over the Christmas/New Year period? | Feedback was unanimous that attempting to collect data over the holiday period would result in lower response rates. | 0 yes, 3 no | *Even if people aren't actively annoyed by being contacted over this period, there is a very good chance they will forget to participate* |
| Newsletters | Newsletters should be:   - Sent by email - Not combined with other messaging (such as reminders, etc.) - Include an opt-out function, if possible | 3 yes, 0 no | N/A |
| Birthday Greetings | Not considered acceptable; likely perceived as annoying spam at best, at worst participant would feel uncomfortable receiving birthday greetings from sexual health researchers. | 0 yes, 3 no | *it might help people be more open if there is a degree of separation between their personal info (birthday) and the messages they receive.* |
| **Timing and nature of survey invitations** | | | |
| Invites by email | See above – preference for all communications by email |  |  |
| Prompts | Mixed opinions on the utility of prompts.    Research team decided to use test kit prompts as an opportunity to check participants’ postal address and include brief mention that questionnaire would arrive by email in a few days. | 2 yes, 1 no | N/A |
| **Value and implementation of financial incentives** | | | |
| Voucher schedule 1 | Month 0 (joining the study) £5  Month 3 kit £10  Month 3 survey £5  Month 6 survey £10  Month 12 kit £25  Month 12 survey £10 | 0 yes, 2 no | - |
| Voucher schedule 2 | Month 0 (joining the study) £5  Month 3 kit £10  Month 3 survey £5  Month 6 survey £10  Month 12 kit £20  Month 12 survey £15 | 3 yes, 0 no | - |
| **Reminders to complete activities** | | | |
| Reminders | A total of 3 reminders was decided upon for the test kits and questionnaires.    Members were in favour of test kit reminders 10 days apart. The research team decided to have the first reminder 3 days after test kit receipt, with the remainder 10 days apart, due to concerns that leaving 10 days might miss vital opportunity to nudge participants to complete their sample and post it back in a timely manner.    Members in favour of a deadline being mentioned in reminders, but research team concerned that that this would discourage completion and instead opted to emphasise the incentives of completion. | 2 yes, 1 no | N/A |
| **Actions to minimise non-response** | | | |
| *No voting; all strategies had mutual agreement from Stages 2 and 3* | | | |
